# Allograft tolerance after adult living donor liver transplantation: a case-control study

**DOI:** 10.1186/s12893-025-02780-5

**Published:** 2025-01-30

**Authors:** Mohamed S. Habl, Moataz Maher Emara, Reham A. Zayed, Ahmed M. Sultan, Ahmed Elsabagh, Ahmed Marwan Elsaid, Ehab E. Abdel-khalek, Mohamed M. El-Saadany, Mohamed Abdel Wahab, Ahmed Shehta

**Affiliations:** 1 Department of Hepatology and Gastroenterology, Faculty of Medicine, Mansoura University, Mansoura, Egypt; 2 Department of Anesthesiology and Intensive Care and Pain Medicine, Faculty of Medicine, Mansoura University, Mansoura, Egypt; 3 Gastrointestinal Surgery Center, Department of Surgery, Faculty of Medicine, Mansoura University, Mansoura, Egypt; 4 Liver Transplantation Program, Gastrointestinal Surgery Center, Faculty of Medicine, Mansoura University, Mansoura, 35511 Egypt

**Keywords:** Living-donor liver transplantation, Allograft tolerance, Immunosuppression, Graft rejection

## Abstract

**Background:**

To investigate the incidence and potential predictors of immune tolerance among adult living donor liver transplant (LDLT) recipients.

**Methods:**

This case-control study included adult recipients who underwent LDLT between May 2004 and January 2018, with at least a 5-year follow-up after LDLT. We divided the study recipients into two groups: Group 1 (Tolerance Group) included recipients who achieved operational or prope tolerance for at least one year; Group 2 (Control Group) included recipients who did not achieve tolerance. We used logistic regression analysis to study the potential predictors of tolerance after LDLT.

**Results:**

We included 368 recipients, 275 (74.7%) in Group 1 and 93 (25.3%) in Group 2. Operational tolerance occurred in 13/275 (4.7%) recipients and prope tolerance in 262/275 (95.3%) recipients. Age was significantly higher in Group 1. The median time for tolerance among the study recipients was 60 months (36–168). During follow-up, Group 1 showed lower serum levels of bilirubin, liver enzymes, alkaline phosphatase, and gamma-glutamyl transferase. Group 1 had a lower incidence of acute cellular rejection (ACR), recurrent viral hepatitis, and biliary complications. Logistic regression identified preoperative MELD, indication for LDLT, ACR, recurrent viral hepatitis, and biliary complications as significant predictors for allograft tolerance after LDLT.

**Conclusion:**

Allograft tolerance occurred in 74.7% of this cohort. We suggest that the MELD score, indication for LT, ACR, recurrent viral hepatitis, and biliary complications are predictors of allograft tolerance after LDLT.

## Background

Liver transplantation (LT) has been recognized as the most effective treatment for irreversible acute or chronic liver failure and selected liver malignancies, with satisfactory results in short- and long-term survival rates [1, 2]. Advances in immunosuppressive drugs, perioperative patient care, and technical aspects have significantly improved LT outcomes.

The overall patient and graft survival rates at one year had risen to nearly 90%. Furthermore, the 10-year overall survival rate has increased to almost 60% [3, 4]. However, the overall life expectancy of liver transplant recipients is lower than that of the general population due to the obligatory use of lifelong immunosuppressive medications [5]. Several adverse effects have been reported to the prolonged use of immunosuppressive medications including nephrotoxicity, neurological complications, cardiovascular diseases, bone abnormalities, opportunistic infections, and de novo neoplasms [5, 6].

Allograft immune tolerance can develop in LT recipients, a phenomenon characterized by the ability to partially (prope tolerance) or completely (operational tolerance) withdraw immunosuppressive medications without graft-related complications [7]. Spontaneous immune tolerance was first described in a key paper by Medawar and his colleagues in 1953, as a specific failure of the recipient’s immunological response [8]. Additionally, studies on experimental transplantation animal models explained the mechanisms of graft tolerance and rejection [9, 10].

In clinical practice, achieving immune tolerance among LT recipients is challenging because allergenic engraftment is not naturally occurring, and graft rejection is the most frequent and potent immunologic response. Preserving the long-term liver graft function has become the primary goal of immunosuppressive medication in recent years, not only preventing frequent acute rejection episodes [11, 12]. Thus, understanding the potential predictors of immune tolerance after LT is of paramount importance.

The current study aims to investigate the frequency of immune tolerance (both prope and operational tolerance) among adult living donor LT (LDLT) recipients and identify potential predictors of tolerance among these patients.

## Methods

### Study design

This is a case-control study based on the prospectively filled medical electronic system records. An informed consent was obtained from each recipient and donor. The present study was approved by the institutional review board, Faculty of Medicine, Mansoura University (IRB Code: 23.11.2383). This manuscript has been reported according to the STROBE statement for case-control studies [13].

We included adult patients (≥ 18 years) who underwent LDLT at the Gastrointestinal Surgery Center, Mansoura University between May 2004 and January 2018, with at least a 5-year follow-up after LDLT.

### Exclusion criteria

1. Death or missing follow-up within 5 years after LT.
2. Recipients with the diagnosis of autoimmune diseases before LT (steroid-dependent therapy).

### Study groups

We divided the study recipients into two groups (Cases and controls) according to the latest immunosuppression medications (level of the trough levels) guided by serum liver function tests.

#### Group 1 (Tolerance Group)

included LT recipients who achieved operational or prope tolerance for at least one year.

#### Group 2 (Control Group)

included LT recipients who did not achieve tolerance.

### Preoperative preparation and surgical technique

Preoperative recipient evaluation protocol and the details of the standardized surgical technique had been described before [14–16].

### Postoperative care and follow-up

After surgery, all recipients were transferred to the intensive care unit, and then to the ward for monitoring graft function. Doppler ultrasound (US) was routinely performed to check liver graft status and patency of the vascular anastomoses, according to the previously described protocol.

After discharge, all recipients were regularly followed up at the outpatient clinic. Follow-up visits included clinical examination, serum laboratory tests including serum trough level of immunosuppression drugs, and Doppler US [6, 14].

### Immunosuppression protocol

#### Immunosuppression protocol after LT

##### Intraoperative immunosuppression

All recipients received intravenous methylprednisolone (500 mg) upon liver graft perfusion. After completion of hepatic artery anastomosis, 500 mg of mycophenolate mofetil (MMF) was administered through the nasogastric tube while basiliximab (20 mg) was administered intravenously. An additional dose of basiliximab was given intravenously on the fourth postoperative day.

##### Postoperative immunosuppression

Double drug therapy was commonly utilized among recipients unless there were contraindications, which include: Tacrolimus, Cyclosporine, or Everolimus + MMF.

For Tacrolimus, we used on all cases if not contraindicated targeting a serum trough level of 7–11 ng/ml during the first 6 months after transplantation, then 5–7 ng/ml after the first 6 months up to 2 years after transplantation, and less than 5 ng/ml thereafter. For Cyclosporine, we used in cases of tacrolimus neurotoxicity or patients with history of neurological diseases which can be aggravated by using tacrolimus targeting a serum trough level of 150–250 ng/ml during the first 6 months after transplantation, then 100–150 ng/ml after the first 6 months up to 2 years after transplantation, and less than 100 ng/ml. After that, if the recipient experienced rising serum creatinine, we decreased the dosage of Tacrolimus or Cyclosporine or discontinued the use of those medications. If the recipient developed cytopenia and did not respond to medical management, we decreased the MMF dosage.

If calcineurin inhibitors (tacrolimus, cyclosporine) could not be used such as recurrent HCC, de novo tumors and chronic kidney disease, we prescribed mTOR inhibitors (Everolimus). Everolimus was used after the first 30 postoperative days if the patient had severe renal impairment, after the pathologic diagnosis of hepatocellular carcinoma, and after the diagnosis of de novo malignancies. Everolimus was given as a starting dose of 1 mg twice a day without a loading dose. The dose is titrated to obtain a serum trough level between 3 and 8 ng/ml during the first year then the dose to be around 3 ng/ml after the first year [6, 17, 18].

The treatment of acute cellular rejection (ACR) depends on time of development of ACR, and histopathological grade of ACR. Time from LT to first post-biopsy-proven acute rejection (BPAR) was categorized as 0–6 (early ACR), > 6 months (late ACR) after LT. The various possible rejection grades based on Banff schema are as follows: a score of 0–2 is no rejection, 3 is borderline (consistent with), 4–5 is mild, 6–7 is moderate and 8–9 as severe ACR. However, higher rejection activity index does not translate into less response to steroids. In mild rejection, we treated by optimization of dose of calcineurin inhibitors to the highest trough level (tacrolimus trough level 11 ng/ml, cyclosporine trough level 250 ng/ml). in moderate rejection, we treated by optimization of dose of calcineurin inhibitors to the highest trough level plus 500 mg pulse of methylprednisolone is given for 1 day followed by tapering the dose.

In severe rejection, we treated by optimization of dose of calcineurin inhibitors to the highest trough level plus 500–1000 mg pulse of methylprednisolone is given for 1–3 days followed by tapering the dose.

#### Immunosuppression minimization protocol after LT

For immunosuppression minimization, we attempted to reduce the dose of MMF after the first year after transplantation and discontinue the drug after the second year or after therapy for the hepatitis C virus (HCV).

We reduced the dose of Tacrolimus to 1–2 mg per day with serum trough level of 7–11 ng/ml during the first 6 months after transplantation, then 5–7 ng/ml after the first 6 months up to 2 years after transplantation, and less than 5 ng/ml thereafter, Cyclosporine to 50–100 mg per day with serum trough level of 150–250 ng/ml during the first 6 months after transplantation, then 100–150 ng/ml after the first 6 months up to 2 years after transplantation, and less than 100 ng/ml, and Everolimus to 0.75 mg per day with serum trough level between 3 and 8 ng/ml during the first year then the dose to be around 3 ng/ml after the first year. All drug minimizations depend on serum trough level of the medications and liver functions with every reduction in the immunosuppression regimen. We did not depend on routine liver biopsy to monitor the immunosuppression withdrawal unless suspicion of graft rejection. Trials for withholding the immunosuppressive medications were attempted five years after transplantation to achieve operational tolerance. We stop drug such as tacrolimus (by gradual withdrawal 1 mg every 3 months till stoppage with monitoring by clinical examination and liver function tests), cyclosporine (by gradual withdrawal 50 mg every 3 months till stoppage with monitoring by clinical examination and liver function tests) and everolimus (by gradual withdrawal 0.75 mg every 3 months till stoppage with monitoring by clinical examination and liver function tests).

### Data collection and study variables

Data from prospectively filled medical electronic system records was collected at the time of the last follow-up visit or 6 months after achieving tolerance. Data included patients’ demographics, clinical and laboratory parameters, and current immunosuppression therapy.

Possible predictors and confounders were then screened based on the clinical data, including age at transplantation, sex, ABO-Rh blood typing, indication of LT, and liver function indicators during the postoperative follow-up period, donors’ characteristics (sex, ABO-Rh blood typing, degree of relationship), and possible operative data including warm ischemic time and cold ischemic time.

We reported laboratory results 6 months after the reduction or discontinuation of immunosuppression drugs in the Tolerance group. While in the non-tolerance group, we reported the last available laboratory results at the last follow-up visit.

The diagnosis of post-LT ACR was diagnosed by two liver biopsies. Recurrent viral hepatitis was diagnosed by a combination of biopsy-based and positive PCR or positive anti-HCV antibodies in patients who received LDLT due to HCV cirrhosis.

### Definition of prope and operational tolerance

Prope tolerance is a condition in which LT recipients with normal laboratory values and stable clinical status are on low-dose immunosuppression monotherapy (Tacrolimus around 3 ng/ml, Cyclosporine level < 100ng/ml, and Everolimus $\:\le\:$ 3ng/ml) without ACR for at least one year [19].

Operational tolerance is a state where the recipient accepts the allograft in the absence of immunosuppression treatment without any abnormality in laboratory values and stable clinical status for at least one year [20].

### Statistical analysis

Numerical data were presented as means (standard deviation) or medians (interquartile ranges) based on the parametric assumptions of data tested using the Shapiro test, histogram, and Q–Q plot. The student’s t-test or Wilcoxon–Mann–Whitney test was used to compare groups as appropriate. Categorical data were depicted as numbers and percentages and compared with the X2 or Fischer’s exact test as appropriate.

We ran a multivariable logistic regression to assess tolerance with multiple predictors, including age at LT (years), birth sex (male/female), the main indication of LT, MELD, recipient-donor relationship (related/unrelated), ABO-Rh compatibility (Yes/No), ACR (Yes/No), post-LT viral hepatitis after LT (Yes/No), and postoperative biliary complications (Yes/No).

The assumptions assessed by the binary logistic regression models were the linearity of the continuous predictors using the Box–Tidwell approach, their independence, the absence of multicollinearity reporting the variance inflation factor (VIF < 4), and residual outliers. We presented the adjusted and unadjusted odds ratios (ORs) in a table, reporting the 95% CI, details of the model, degree of freedom (df), and R2 (the coefficient of multiple determination of the model).

Statistical analysis was performed using the IBM-SPSS statistical package (SPSS Inc., Chicago, USA, version 27), with a two-tailed P value of ≤ 0.05 indicating statistical significance.

## Results

**Fig. 1:**
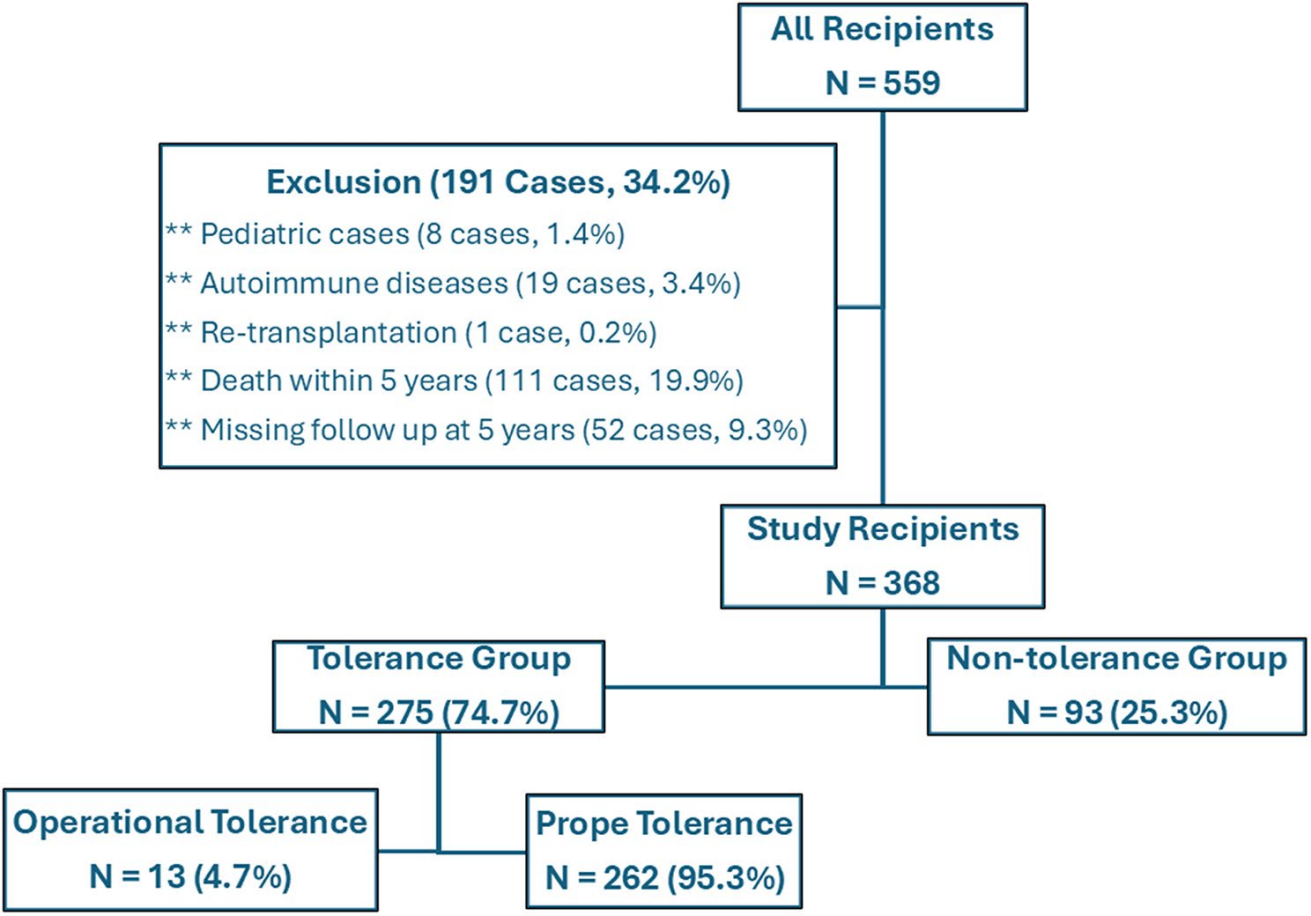
Flow chart of the study recipients

Out of 559 patients who underwent LDLT during the study period, we included 368 (Fig. 1). The Tolerance Group included 275 recipients (74.7%), while the non-Tolerance Group included 93 recipients (25.3%). Operational tolerance occurred in 13/275 (4.7%) recipients and prope tolerance in 262/275 (95.3%) recipients (Fig. 1).

### Recipient’s characteristics

The recipient’s characteristics are summarized in Table 1. Age was significantly higher in the Tolerance Group. The main indications for liver transplantation were HCV-related cirrhosis and hepatocellular carcinoma.

**Table 1:** Pre-LT recipients’ characteristics, presented as no. (%), mean (SD), and median (Q1-Q3)

	Tolerance Group (*N* = 275)	Non-Tolerance Group (*N* = 93)	*P value*
**Age at LT (years)**	50.4 (6.7)	48.2 (7.3)	0.009*
**Pre-LT BMI (kg/m2)**	29.1 (4)	29.7 (4.4)	0.203
**Male**	249 (90.5%)	86 (92.5%)	0.574
**Pre-LT MELD**	15.35 (4.6)	16.65 (6.16)	0.033*
**Indication of LT**			0.154
HCV cirrhosis HCC Others	163 (59.3%) 102 (37%) 10 (3.6%)	51 (54.8%) 34 (36.6%) 8 (8.6%)	
**Cold ischemia time (min)**	31 (24–43)	35 (25–45)	0.098
**Warm ischemia time (min)**	38 (32–46)	37 (33–55)	0.232

*Abbreviations*** LT**: Liver Transplantation, **SD**: standard deviation, **Q**: Quartile, **BMI**: Body Mass Index, **MELD**: Model for End-stage Liver Disease, **HCV**: Hepatitis C Virus, **HCC**: Hepatocellular Carcinoma

**Other indications of LT were** Hepatitis B virus cirrhosis, Welson’s disease, Cryptogenic cirrhosis, and Budd-Chiari Syndrome

(*) indicates statistical significance (*P* < 0.05)

### Donor’s characteristics

As shown in Table 2, donors did not show significant differences between the two groups regarding donor age, sex, relationship, and ABO-Rh blood group typing.

**Table 2:** Pre-LT characteristics of donors and Recipient/Donor relationship, *presented as mean (SD) and no. (%)*

	Tolerance Group (*N* = 275)	Non-Tolerance Group (*N* = 93)	*P-value*
Age (years)	27.3 (5.4)	28.4 (6.8)	0.129
Male	194 (70.5%)	70 (75.3%)	0.357
Donor Genetic Relation			0.546
Unrelated 1st degree 2nd degree 3rd degree 4th degree	58 (21.1%) 119 (43.3%) 34 (12.4%) 44 (16%) 20 (7.3%)	27 (29%) 36 (38.7%) 12 (12.9%) 11 (11.8%) 7 (7.5%)	
Related donors (Binary)	217 (78.9%)	66 (71%)	0.116
Identical ABO blood groups	199 (72.4%)	74 (79.6%)	0.17
Identical ABO-Rh groups	178 (64.7%)	63 (67.7%)	0.597
Identical Rh groups	249 (91.2%)	82 (88.2%)	0.39

*Abbreviations*** SD**: standard deviation, **LT**: Liver Transplantation

### Immunosuppressive medications on follow-up

The median time for tolerance among the study recipients was 60 months (36–168). A summary of the immunosuppressive medications at the time of the study is shown in Table 3. The tolerance group showed the required trough levels of immunosuppressive medications for confirming allograft tolerance.

**Table 3:** Immunosuppression and laboratory results on follow-up, *presented as no. (%) and median (Q1-Q3)*

	Tolerance Group (*N* = 275)	Non-Tolerance Group (*N* = 93)	*P*-value
Tacrolimus	187 (68%)	70 (75.3%)	0.187
Tacrolimus trough level (ng/ml)	3 (2.15–3.7)	4.4 (3.2–6.3)	< 0.001*
Cyclosporine	39 (14.2%)	6 (6.5%)	0.049*
Cyclosporine trough level (ng/ml)	64 (45–92.8)	127.7 (64.1– 127.7)	0.038*
Everolimus	16 (17.2%)	21 (7.6%)	0.008*
Everolimus trough level (ng/ml)	1.8 (1.1–3)	1.9 (1.35–2.9)	0.868
Mycophenolate mofetil	15 (5.5%)	32 (34.4%)	< 0.001*
Serum creatinine (mg/dl)	1.1 (0.9–1.3)	1.1 (0.9–1.5)	0.049*
Serum albumin (g/dl)	4.5 (4.2–4.7)	4.4 (4–4.7)	0.006*
Serum total bilirubin (mg/dl)	0.7 (0.5–0.9)	0.8 (0.6–1.3)	< 0.001*
Serum direct bilirubin (mg/dl)	0.1 (0.1–0.1)	0.1 (0.1–0.6)	< 0.001*
AST (IU/L)	22 (21–27.5)	36 (26–57)	< 0.001*
ALT (IU/L)	23 (20–29.5)	39 (27–61)	< 0.001*
ALP (KAU)	5 (5–6)	8 (6–16)	< 0.001*
Serum GGT (IU/L)	34 (22–54)	177 (102–307)	< 0.001*

*Abbreviations*** AST**: Aspartate Transaminase, **ALT**: Alanine Transaminase, **ALP**: Alkaline Phosphatase, **GGT**: Gamma-Glutaryl Transferase

(*) indicates statistical significance (*P* < 0.05)

### Laboratory results on follow-up

The tolerance group showed lower serum levels of total bilirubin, direct bilirubin, AST, ALT, ALP, and GGT (Table 3).

### Postoperative complications

Patients with tolerance had a lower incidence of ACR (by liver biopsy), recurrent viral hepatitis, and biliary complications (requiring intervention) (Table 4).

**Table 4:** Postoperative complications, *presented as no. (%)*

	Tolerance Group (*N* = 275)	Non-Tolerant Group (*N* = 93)	*P-value*
**ACR**	114 (41.5%)	63 (67.7%)	< 0.001*
**ACR frequency**			< 0.001*
0 1 2 3 4 6	161 (58.5%) 83 (30.2%) 22 (8%) 8 (2.9%) 1 (0.4%) 0	30 (32.3%) 33 (35.5%) 20 (21.5%) 6 (6.5%) 3 (3.2%) 1 (1.1%)	
**Recurrent viral hepatitis**	62 (22.5%)	37 (39.8%)	0.001*
**Recurrent viral hepatitis frequency**			0.001*
**0** **1** **2** **3** **4** **5**	213 (77.5%) 50 (18.2%) 9 (3.3%) 2 (0.7%) 0 1 (0.4%)	56 (60.2%) 22 (23.7%) 10 (10.8%) 4 (4.3%) 1 (1.1%) 0	
**Biliary Complications**	72 (26.2%)	50 (53.8%)	< 0.001*

*Abbreviations*** ACR**: Acute Cellular Rejection

(*) indicates statistical significance (*P* < 0.05)

### Regression analysis of allograft tolerance

The binary multivariable logistic regression model showed statistical significance [X2 (df = 10) = 64.95, *P* < 0.001], explaining 23.9% (Nagelkerke R2) of the variance in allograft tolerance and correctly diagnosing 79.6% of the cases (Table 5). Preoperative MELD, indication for LT (HCV-related cirrhosis versus others), ACR, post-LT viral hepatitis, and biliary complications were significant predictors for allograft tolerance after living-donor LT (Table 5).

**Table 5:** Regression analysis for predictive factors for tolerance

	Unadjusted OR (95% CI)	*P-value*	Adjusted OR (95% CI)	*P-value*
Age at LT	1.04 (1.01–1.08)	0.01*	1.034 (0.99–1.08)	0.082
Male	1.283 (0.54–3.06)	0.575	1.7 (0.61–4.76)	0.308
Pre-LT MELD	0.95 (0.91–0.997)	0.037*	0.95 (0.9–0.99)	0.033*
Related donation	1.53 (0.89–2.61)	0.118	1.23 (0.68–2.24)	0.492
ABO-Rh compatibility	0.87 (0.53–1.44)	0.597	0.81 (0.46–1.46)	0.459
Diagnosis HCV/HCC	0.94 (0.57–1.55)	0.804	0.62 (0.35–1.09)	0.096
Diagnosis HCV/Others	0.39 (0.15–1.4)	0.06	0.23 (0.08–0.72)	0.011*
ACR	0.34 (0.21–0.55)	< 0.001*	0.36 (0.21–0.62)	0.001*
Post-LT viral hepatitis	0.44 (0.27–0.73)	0.001*	0.44 (0.25–0.78)	0.005*
Biliary complications	0.31 (0.19–0.5)	< 0.001*	0.28 (0.16–0.48)	0.001*

*Abbreviations*** OR: LT**: Liver Transplantation, **MELD**: Model for End-stage Liver Disease, **HCV**: Hepatitis C Virus, **HCC**: Hepatocellular Carcinoma, **ACR**: Acute Cellular Rejection. **Others**: refer to Hepatitis B virus cirrhosis, Welson’s disease, Cryptogenic cirrhosis, and Budd-Chiari Syndrome

(*) indicates statistical significance (*P* < 0.05)

## Discussion

Allograft tolerance after LDLT represents a promising avenue for improving transplant outcomes and minimizing side effects associated with long-term immunosuppression drugs use. In this case-control study, spontaneous allograft tolerance occurred in 275 LDLT recipients (74.7%) with a minimal follow-up of 5 years. Of those, operational tolerance occurred in 13 (4.7%) recipients and prope tolerance in 262 (95.3%) recipients. The risk factors for failure of development of tolerance were the higher pre-LT MELD, post-LT ACR, post-LT viral hepatitis, and post-LT biliary complications. Multivariable regression analysis confirmed these factors in addition to the primary indication of LT can predict tolerance after LT.

Recipients with higher age at the time of LT were more likely to develop tolerance. Increasing the frequency of ACR and recurrent viral hepatitis were associated with increasing incidence of non-tolerance. Unlike in our sample (allograft tolerance is 74.7%), other studies reported that 20–40% of liver transplant recipients can sustain graft tolerance [21, 22]. Other reports showed that more than 30% of selected adult recipients and 60% of selected pediatric recipients of LT could effectively achieve operational tolerance [23–26]. This might be attributed to different demographic characteristics, underlying liver pathology, immunosuppression protocol, and postoperative complications in the different studies.

Immune tolerance after LT can be therapeutic or spontaneous. Therapeutic immune tolerance is the pharmaceutical induction of immune tolerance [10, 22]. Spontaneous immune tolerance is described as operational tolerance or prope tolerance, depending on the ability to withdraw or reduce immunosuppression medications. However, this phenomenon is still not well understood. Currently, there is no general agreement on parameters to indicate the possibility for immunosuppression withdrawal and the duration of maintenance of stable liver functions to define spontaneous immune tolerance [27–29].

As shown in Table 3, the trough level of immunosuppression drugs was lower in the Tolerance group, confirming the chosen tolerance definition. A greater proportion of patients in the Tolerance group were treated with Everolimus, likely due to three proposed mechanisms: it prevents the endocytosis of antigens by dendritic cells, inhibiting their full maturation into antigen-presenting cells [2, 30]; it enhances the activity of regulatory T cells, which reduces T cell migration to the transplanted liver, thereby lowering the risk of rejection [3, 31]; and it limits the formation of memory cells, fostering a tolerance-promoting environment [32].

Likewise, developing Tolerance was associated with taking Cyclosporine (Table 3). Cyclosporine blocks apoptosis of activated T cells creating peripheral transplant tolerance. Second, it hinders IL-2 gene expression and activation-induced cell death of alloreactive T effector cells. Third, cyclosporin enhances the T regulatory cell function and the allograft tolerance by abolishing donor-specific transfusion (anti-CD154) [33]. In contrast, a larger proportion of patients receiving MMF were in the non-tolerant group (34.4% vs. 5.5%). This may be attributed to that MMF is usually used for treating rejection, especially in steroid-resistant acute rejection while continuing the main therapy [34].

A higher serum creatinine was observed in the non-tolerant group (Table 3). The higher doses of tacrolimus in the non-tolerant group and the associated nephrotoxicity may explain this finding [35]. In addition, the higher GGT and serum bilirubin in the non-tolerant group (Table 3) represent the cholestasis-associated acute or chronic rejection [36]. However, we suggest the GGT may be more sensitive to represent the non-tolerance than bilirubin as the difference was more prominent in GGT serum levels (Table 3).

The major proportion of the living donors in the current study were genetically related to their recipients with no differences between either group (78.9% in tolerance vs. 71% in the non-tolerance group, *P* = 0.116). Our previous study showed that recipients with genetically related liver donors experience a lowered incidence of rejection episodes when compared to non-genetically related liver donors [18].

Although there is a notable age difference between the recipients, it is small and clinically irrelevant. Furthermore, the tolerance group exhibited slightly better follow-up laboratory results, though values were nearly normal in both groups, with the exception of GGT, which was abnormally elevated in the non-tolerance group at 177 IU/L (102–307). This may suggest that recipients who develop tolerance tend to have better liver graft quality. Such recipients also typically need fewer immunosuppressive medications, experience fewer drug-related complications, and achieve a better quality of life [37].

We found that post-LT ACR was associated with less likelihood of developing tolerance (41% in tolerance groups versus 67.7% in the non-tolerance group, *P* < 0.001). Additionally, the more frequent the ACR is, the less likely the tolerance happens, *P* < 0.001. Liver graft rejection, including ACR, is one of the most frequent complications after LT, hence, many LT recipients may require life-long immunosuppression medications [6, 38]. Both memory T-cells of the CD4 + helper class and CD8 + memory T-cells play essential roles in graft rejections. Memory T-cells of the CD4 + helper class induce antibody-mediated rejection via enhanced production of donor-specific antibodies by B-cells. CD8 + memory T-cells can exert direct cytotoxic effects. Compared to naïve T-cells, memory T-cells are less susceptible to immunosuppressive therapies, which may account for some patients’ partial response to conventional therapies for acute cell-mediated rejection [38, 39]. Therefore, memory T-cells could be a barrier to developing immune tolerance, however, more research is needed to clarify such findings.

The current study showed a higher incidence of biopsy-proven recurrent HCV hepatitis among the non-tolerant than tolerant group; 37 cases (39.8%) versus 62 cases (22.5%), *P* = 0.001. Additionally, the more frequent HCV hepatitis is, the less likely the tolerance happens, *P* = 0.001. previously, our group reported that HCV hepatitis recurred in 90.3% of recipients who underwent LDLT due to HCV-associated end-stage liver disease (ESLD). Older donor age and prolonged warm ischemia time are independent predictors of HCV recurrence after LT [40]. Grassi and colleagues indicated that CD4- and CD8 T-cells are involved in post-LT HCV recurrence. The CD8 T-cell subset makes up most intrahepatic lymphocytes in patients with recurrent HCV following liver transplantation [41]. Rosen and colleagues showed the presence of HCV-specific MHC class II-restricted CD4 T-cell responses in patients with HCV recurrence after LT even with immunosuppression medications [42]. Therefore, HCV recurrence exaggerates the immune response among LT recipients and may lower the chance of developing immune tolerance.

Biliary complications were significantly prevalent in the non-tolerance group (53%) when compared to the tolerance group (26.2%), *P* < 0.001. In our center, we found that biliary complications account for almost 30% of LT recipients including bile leakage, biloma, and biliary structures [43]. The immunological traits of recipients with biliary complications are still unclear. Lei and colleagues found that LT recipients with biliary complications experienced a decrease in CD4 + T cells, naïve T cells, and stem cell memory T cells. In contrast, CD8 + T and effector memory T cells in CD8 + T cells increased [44]. Additionally, biliary complications and pathogenic infections were found to increase the risk of antigen exposure and rejection episodes [45].

Our study has some limitations. First, it is a retrospective single-center study with a risk of selection bias. Second, the sample size is small with HCV-related ESLD was the main indication for LT. Third, we did not depend on routine follow up liver biopsy for monitoring the recipients during immunosuppression withdrawal. However, the current study is the first to evaluate the prevalence and predictors of immune tolerance among adult LDLT recipients in the locality. Future multicenter and prospective studies are essential to confirm and further study the predictors and characteristics of tolerance in LDLT.

In conclusion, we found that preoperative MELD, indication for LT, ACR, post-LT viral hepatitis, and biliary complications may predict allograft tolerance after LDLT.

## Data Availability

The data generated and analyzed for the current manuscript is not publicly available and will be available by a reasonable request from the corresponding author.
